# Automated machine learning for predicting liver metastasis in patients with gastrointestinal stromal tumor: a SEER-based analysis

**DOI:** 10.1038/s41598-024-62311-9

**Published:** 2024-05-30

**Authors:** Luojie Liu, Rufa Zhang, Ying Shi, Jinbing Sun, Xiaodan Xu

**Affiliations:** 1 Department of Gastroenterology, Changshu Hospital Affiliated to Soochow University, Suzhou, China; 2 Department of General Surgery, Changshu Hospital Affiliated to Soochow University, Suzhou, China

**Keywords:** Automated machine learning, Liver metastasis, Gastrointestinal stromal tumors, SEER, Cancer, Cancer models

## Abstract

Gastrointestinal stromal tumors (GISTs) are a rare type of tumor that can develop liver metastasis (LIM), significantly impacting the patient's prognosis. This study aimed to predict LIM in GIST patients by constructing machine learning (ML) algorithms to assist clinicians in the decision-making process for treatment. Retrospective analysis was performed using the Surveillance, Epidemiology, and End Results (SEER) database, and cases from 2010 to 2015 were assigned to the developing sets, while cases from 2016 to 2017 were assigned to the testing set. Missing values were addressed using the multiple imputation technique. Four algorithms were utilized to construct the models, comprising traditional logistic regression (LR) and automated machine learning (AutoML) analysis such as gradient boost machine (GBM), deep neural net (DL), and generalized linear model (GLM). We evaluated the models' performance using LR-based metrics, including the area under the receiver operating characteristic curve (AUC), calibration curve, and decision curve analysis (DCA), as well as AutoML-based metrics, such as feature importance, SHapley Additive exPlanation (SHAP) Plots, and Local Interpretable Model Agnostic Explanation (LIME). A total of 6207 patients were included in this study, with 2683, 1780, and 1744 patients allocated to the training, validation, and test sets, respectively. Among the different models evaluated, the GBM model demonstrated the highest performance in the training, validation, and test cohorts, with respective AUC values of 0.805, 0.780, and 0.795. Furthermore, the GBM model outperformed other AutoML models in terms of accuracy, achieving 0.747, 0.700, and 0.706 in the training, validation, and test cohorts, respectively. Additionally, the study revealed that tumor size and tumor location were the most significant predictors influencing the AutoML model's ability to accurately predict LIM. The AutoML model utilizing the GBM algorithm for GIST patients can effectively predict the risk of LIM and provide clinicians with a reference for developing individualized treatment plans.

## Introduction

Within the gastrointestinal tract, gastrointestinal stromal tumors (GISTs) are the most commonly encountered type of mesenchymal tumor^1^. As GISTs have the potential to be malignant^2^, previous study has indicated that more than half of patients with these tumors experience metastasis by the time they seek medical attention, with the liver being the most frequently affected location^3–5^. While the emergence of tyrosine kinase inhibitors (TKIs) such as imatinib has improved survival outcomes in patients with metastatic GIST^6,7^, secondary mutations and drug resistance may occur during adjuvant TKIs treatment, resulting in poor long-term outcomes for patients^8^.

Meanwhile, in the initial stages, numerous GIST patients present with no overt symptoms, and the liver metastases (LIM) can often be indistinguishable from other hepatic conditions on radiographic imaging, compounding the complexity of clinical diagnosis. The standard methods for screening LIM in patients with GISTs are magnetic resonance imaging (MRI) and positron emission tomography/computed tomography (PET/CT)^9^. Nonetheless, due to the high expense associated with MRI and the potential radiation harm from PET-CT, it is not advisable to use these techniques for LIM screening in all GIST patients.

Therefore, constructing a predictive model to accurately forecast the occurrence of LIM in patients with GIST is essential for guiding treatment decisions and improving patient prognosis. Wu et al.^10^ developed a nomogram to forecast the likelihood of distant metastases in patients with GISTs. Meanwhile, Zhou et al.^11^ employed a nomogram to predict the risk of LIM in GISTs patients. However, these traditional nomogram models are limited to linear regression approaches, constraining their capacity to handle complex and non-linear relationships. Furthermore, they do not consistently select the most predictive features during the feature selection process.

Automated machine learning (AutoML) is an advanced branch of machine learning (ML) known for enhancing prediction accuracy while saving time and effort^12,13^. While traditional ML demands deep expertise and ongoing fine-tuning, AutoML simplifies this by automating tasks like feature engineering and hyperparameter tuning. This speeds up modeling and broadens ML's accessibility. Leveraging AutoML models, clinicians can enhance the accuracy of their predictions regarding LIM development in GIST patients. However, no study have applied AutoML to predict LIM in GIST patients to date. To fill this gap, this study developed AutoML models using the Surveillance, Epidemiology, and End Results (SEER) database to predict LIM in GIST patients.

## Materials and methods

### Study population

**Figure 1:**
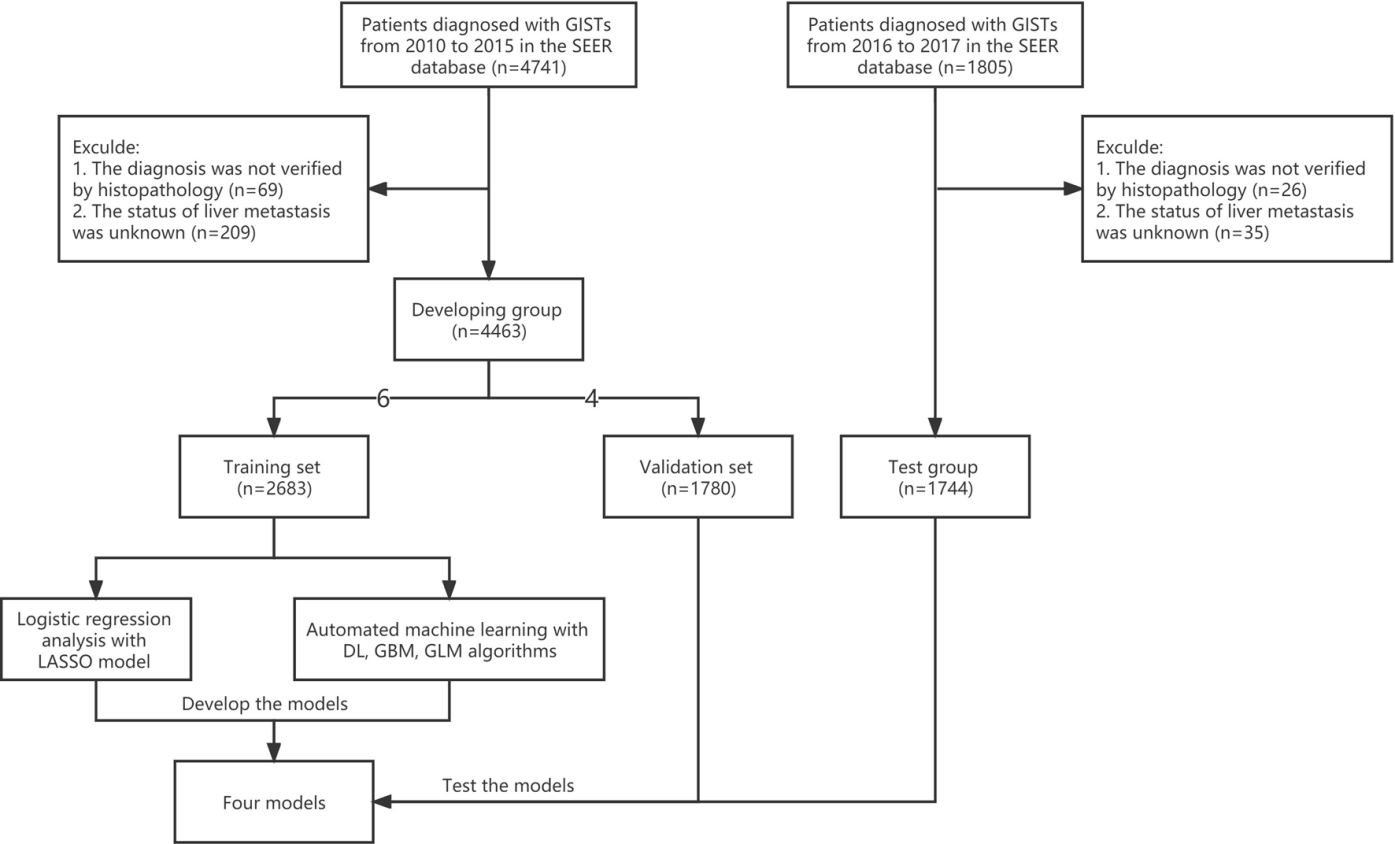
Flow chart of the study. *GISTs* gastrointestinal stromal tumors, *SEER* Surveillance, Epidemiology, and End Results, *GBM* gradient boost machine, *DL* deep neural net, *GLM* generalized linear model, *LASSO* least absolute shrinkage and selection operator.

A retrospective analysis was performed on the SEER database (http://seer.cancer.gov), which is estimated to cover around 28% of the US population and contains comprehensive information on various clinical and pathological aspects of multiple cancers. The identification of GISTs was based on their ICD-O-3 code (8936). Given that the SEER database began providing information on the specific sites of metastatic GIST starting in 2010^14^, we used the SEER*Stat software to select patient data from 2010 to 2017 and then sequentially divided this data into three sets. The training and validation sets were constructed using data from 2010 to 2015, while the test set was constructed using data from 2016 to 2017. We excluded the following patients: (1) The diagnosis was not verified by histopathology; (2) The status of liver metastasis was unknown. A flow chart depicting the study protocol is presented in Fig. 1. The presence of LIM in newly diagnosed GIST patients was determined using "SEER Combined Mets at DX-liver (2010Age was divided into two groups: ≤ 65 years (young group) and > 65 years (elderly group)^15^. The N stages were determined in accordance with the 7th edition of the American Joint Committee on Cancer (AJCC) Cancer Staging Manual^16^. Race was categorized into 3 groups: white, black, and others (including American Indian, Alaska Native and Asian/Pacifc Islander). The tumor location was categorized into 4 groups: stomach, small intestine, colon, and others (tumor locations with fewer than 20 reported cases primarily involve extragastrointestinal stromal tumors). Marital status was divided into two categories: married and unmarried (which includes divorced, separated, single, and widowed). The variable "CS site-specific factor 6" was utilized for the identification of the mitotic rate. The Institutional Review Board did not require approval for the study because SEER includes patient information that cannot be used to identify individuals.

### Multiple imputation

Owing to the presence of missing data in several variables such as race (1.0% of cases), tumor size (7.8% of cases), N stage (6.4% of cases), marital status (5.4% of cases), and mitotic rate (29.9% of cases), we employed a polytomous regression model using the multiple imputation (MI) feature in R software (version 4.1.0). This method was adopted to bolster the study's analytical robustness.

### Logistic regression analysis

To address the problem of multiple collinear relationships among the explanatory variables, we conducted a univariate analysis using the least absolute shrinkage and selection operator (LASSO) regression model with the "λ" criterion. Subsequently, a binary logistic backward stepwise regression analysis was employed to further refine the model. The performance of the resulting model was assessed by calculating the area under the receiver operating characteristic curve (AUC), calibration curve, and decision curve analysis (DCA). DCA is a tool designed to evaluate the clinical benefits of predictive models. It primarily determines whether the application of a model at various probability thresholds offers advantages over blanket treatment strategies or opting for no treatment at all. The DCA curve illustrates the net benefit of the predictive model at different probability thresholds, aiding clinicians in better balancing risks and rewards during the decision-making process^17^. In addition, a nomogram, serving as a visual tool to translate the logistic regression model into an intuitive representation, was developed based on the independent risk factors identified in the multivariate analysis.

### Automated machine learning

AutoML analysis was performed using the H2O package (version 3.42.0.3, 2023 release), which was installed from the H2O.ai platform (www.h2o.ai). H2O.ai stands as a premier platform dedicated to machine learning and artificial intelligence, crafted by the reputed company, H2O.ai. Renowned for its outstanding computational speed, adaptability, and intuitive design, it's a favored choice in the open-source community. A notable feature of the AutoML process in H2O is that it automatically selects appropriate algorithms and combines them into several ensemble models. The set of algorithms consisted of a randomized grid of Gradient Boosting Machines (GBMs), a randomized grid of Deep Neural Networks (DLs), and a fixed grid of Generalized Linear Models (GLMs). GBM, an ensemble method building successive decision trees to correct prior errors, excels in capturing complex patterns and offers high accuracy, making it apt for tasks like disease prognosis and risk stratification^18^. Meanwhile, GLM, which extends linear regression to accommodate non-normal response variables, stands out due to its interpretability, crucial for understanding clinical predictor-response relationships, and is widely employed in epidemiology to gauge predictors' effect on outcomes^19^. On the other hand, DL, utilizing multi-layered neural networks, shines in processing large datasets, particularly medical images and unstructured data, revolutionizing tasks such as disease diagnosis from X-rays and MRIs^20^. Hyperparameter optimization was carried out via a fivefold cross-validation grid search on the training set, evaluating various combinations of hyperparameters included in the grid search based on their AUCs. AutoML visualization was achieved through feature importance, SHAP, and LIME techniques. Using SHAP analysis, we were able to identify the critical features that significantly influenced the model predictions and their contribution to the overall model performance for a specific prediction^21^. LIME analysis showcased the contribution of each feature in predicting the outcome by randomly sampling instances from the validation set and the test set^22^. SHAP and LIME are both techniques used for model interpretability, but they differ in foundational approach and scope. SHAP, rooted in game theory, calculates feature contributions for predictions based on the Shapley value, ensuring a fair distribution among features and providing both global and local interpretability. On the other hand, LIME focuses on local explanations by perturbing the data and fitting an interpretable model to approximate black-box predictions.

### Statistical analysis

In our study, we used the confusion matrix, which includes True Positives (TP), False Positives (FP), False Negatives (FN), and True Negatives (TN), to assess the performance of our classification model. True Positives (TP) represent the cases correctly identified as positive, while False Positives (FP) are the cases incorrectly identified as positive. False Negatives (FN) are cases incorrectly identified as negative, and True Negatives (TN) are the cases correctly identified as negative. To accurately assess the performance of our model, we computed several vital metrics. Sensitivity (or Recall) quantifies how effectively the model identifies actual positives, with its formula being TP/(TP + FN). Specificity evaluates the model's ability to correctly recognize actual negatives, calculated as TN/(TN + FP). The Positive Predictive Value (PPV) measures the accuracy of positive predictions, computed as TP/(TP + FP). Conversely, the Negative Predictive Value (NPV) gauges the accuracy of negative predictions, determined as TN/(TN + FN). Accuracy (ACC) reflects the model's overall correctness in classification, calculated using the formula TP + TN/(TP + TN + FP + FN). Lastly, the F1-Score, serving as the harmonic mean of PPV (Precision) and Sensitivity, offers a balanced measure of these two aspects, computed as 2 × PPV × Sensitivity/(PPV + Sensitivity). Categorical variables were presented as frequencies and percentages, and statistical comparisons between groups were performed using either the Chi-square test or Fisher exact test, depending on the sample size and distribution of data. The statistical significance level was set at *P*  < 0.05 to determine the presence of significant differences between groups. All statistical analyses were conducted using the R software (version 4.1.0) to ensure accuracy and reproducibility of the results.

## Results

### Baseline characteristics of patients

A total of 6207 patients were included in this study. LIM was present in 662 cases, representing 10.7% of the entire cohort. The developing dataset consisted of cases from 2010 to 2015, which were divided into training and validation cohorts in a 6:4 ratio (n = 2683 in the training group and n = 1780 in the validation group). The testing cohort comprised 1744 cases that met the inclusion criteria of being from the years 2016 to 2017. Among the 4463 patients in the developing group, LIM was present in 476 cases (10.7%), while in the testing group, there were 186 cases (10.7%) of LIM out of a total of 1744 cases. Detailed baseline characteristics of patients are listed at Table 1, whereas Supplementary Table 1 offers a detailed breakdown of the demographic and clinical features of the two cohorts before MI. According to Table 2, the correlation analysis showed that in both the developing and testing groups, four clinical characteristics (sex, N stage, tumor size, and mitotic rate) were significantly correlated (*P*  < 0.05) with LIM.

**Table 1:** Baseline characteristics of patients after multiple imputation.

Variables	Entire cohort (n = 6207)	Developing cohort (n = 4463)	Test cohort (n = 1744)	*P*-value
Age, years, n (%)				0.283
≤ 65	3221 (51.9)	2335 (52.3)	886 (50.8)	
> 65	2986 (48.1)	2128 (47.7)	858 (49.2)	
Race, n (%)				0.476
White	4210 (67.8)	3038 (68.1)	1172 (67.2)	
Black	1141 (18.4)	804 (18.0)	337 (19.3)	
Others	856 (13.8)	621 (13.9)	235 (13.5)	
Sex, n (%)				0.866
Male	3196 (51.5)	2301 (51.6)	895 (51.3)	
Female	3011 (48.5)	2162 (48.4)	849 (48.7)	
Location, n (%)				0.934
Stomach	3833 (61.8)	2748 (61.6)	1085 (62.2)	
Small intestine	1602 (25.8)	1162 (26.0)	440 (25.2)	
Colon	290 (4.7)	208 (4.7)	82 (4.7)	
Others	482 (7.8)	345 (7.7)	137 (7.9)	
N stage, n (%)				0.997
N0	5972 (96.2)	4294 (96.2)	1678 (96.2)	
N1	235 (3.8)	169 (3.8)	66 (3.8)	
Tumor size, cm, n (%)				0.006
≤ 2.0	868 (14.0)	584 (13.1)	284 (16.3)	
2.0—5.0	1945 (31.3)	1399 (31.3)	546 (31.3)	
5.0—10.0	1850 (29.8)	1338 (30.0)	512 (29.4)	
> 10.0	1544 (24.9)	1142 (25.6)	402 (23.1)	
Mitotic rate, HPF, n (%)				< 0.001
< 5/50	4222 (68.0)	2977 (66.7)	1245 (71.4)	
≥ 5/50	1985 (32.0)	1486 (33.3)	499 (28.6)	
Marital status, n (%)				0.788
Married	3686 (59.4)	2655 (59.5)	1031 (59.1)	
Unmarried	2521 (40.6)	1808 (40.5)	713 (40.9)	
Liver metastasis, n (%)				1.000
Yes	662 (10.7)	476 (10.7)	186 (10.7)	
No	5545 (89.3)	3987 (89.3)	1558 (89.3)	

*HPF* high power field, *Others (Race)* American Indian, Alaska Native, Asian/Pacifc Islander, *Others (Location)* Tumor locations with fewer than 20 reported cases exist.

**Table 2:** Comparison between patients with liver metastasis and those without metastasis in the developing and testing groups after multiple imputation.

Variables	The developing group (n = 4463)			The test group (n = 1744)		
	Positive (n = 476)	Negative (n = 3987)	*P*-value	Positive (n = 186)	Negative (n = 1558)	*P*-value
Age, years, n (%)			0.246			0.007
≤ 65	261 (54.8)	2074 (52.0)		77 (41.4)	809 (51.9)	
> 65	215 (45.2)	1913 (48.0)		109 (58.6)	749 (48.1)	
Race, n (%)			0.100			< 0.001
White	318 (66.8)	2720 (68.2)		153 (82.3)	1019 (65.4)	
Black	101 (21.2)	703 (17.6)		18 (9.7)	319 (20.5)	
Others	57 (12.0)	564 (14.1)		15 (8.1)	220 (14.1)	
Sex, n (%)			< 0.001			< 0.001
Male	285 (59.9)	2016 (50.6)		151 (81.2)	744 (47.8)	
Female	191 (40.1)	1971 (49.4)		35 (18.8)	814 (52.2)	
Location, n (%)			< 0.001			0.340
Stomach	252 (52.9)	2496 (62.6)		108 (58.1)	977 (62.7)	
Small intestine	106 (22.3)	1056 (26.5)		48 (25.8)	392 (25.2)	
Colon	16 (3.4)	192 (4.8)		13 (7.0)	69 (4.4)	
Others	102 (21.4)	243 (6.1)		17 (9.1)	120 (7.7)	
N stage, n (%)			< 0.001			< 0.001
N0	421 (88.4)	3873 (97.1)		165 (88.7)	1513 (97.1)	
N1	55 (11.6)	114 (2.9)		21 (11.3)	45 (2.9)	
Tumor size, cm, n (%)			< 0.001			< 0.001
≤ 2.0	25 (5.3)	559 (14.0)		6 (3.2)	278 (17.8)	
2.0–5.0	71 (14.9)	1328 (33.3)		26 (14.0)	520 (33.4)	
5.0–10.0	160 (33.6)	1178 (29.5)		60 (32.3)	452 (29.0)	
> 10.0	220 (46.2)	922 (23.1)		94 (50.5)	308 (19.8)	
Mitotic rate, HPF, n (%)			< 0.001			< 0.001
< 5/50	259 (54.4)	2718 (68.2)		103 (55.4)	1142 (73.3)	
≥ 5/50	217 (45.6)	1269 (31.8)		83 (44.6)	416 (26.7)	
Marital status, n (%)			0.420			0.641
Married	275 (57.8)	2380 (59.7)		107 (57.5)	924 (59.3)	
Unmarried	201 (42.2)	1607 (40.3)		79 (42.4)	634 (40.7)	

*HPF* high power field, *Others (Race)* American Indian, Alaska Native, Asian/Pacifc Islander, *Others (Location)* Tumor locations with fewer than 20 reported cases exist.

### Univariate and multivariate logistic regression analysis

**Figure 2:**
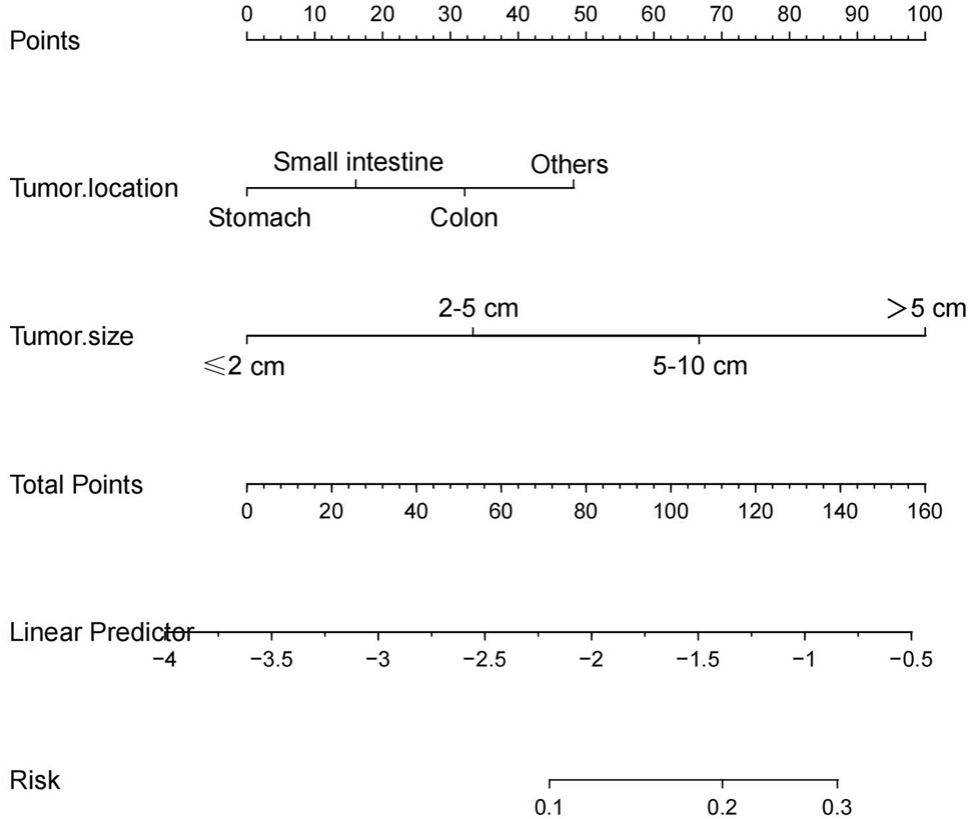
Nomogram of the LASSO model for predicting liver metastasis in patients with gastrointestinal stromal tumor. *HPF* high power field.

The "λ" criterion obtained through fivefold cross-validation was utilized to apply the LASSO regression model, resulting in the selection of two out of eight variables as independent risk factors. Supplementary Fig. 1 illustrates that this method was utilized to resolve the issue of multiple collinear relationships among the explanatory variables. The final logistic model, which included two variables (tumor location and tumor size), was presented in the form of a nomogram and a score system, making it appropriate for clinical use (Fig. 2). Supplementary Fig. 2 illustrates the calibration curves for the training, validation, and test sets, which had mean absolute errors of 0.012, 0.008, and 0.003, respectively. These calibration curves indicate that the estimated risk produced by the LASSO model closely approximated the actual risk, suggesting a high level of reliability. According to the DCA plots for the LASSO model in the training set, validation set and test set, Supplementary Fig. 3 shows that if the threshold probability for predicting LIM by the LASSO model falls between 10 and 40%, an intervention may result in a benefit of 7%.

### Automated machine learning analysis

**Figure 3:**
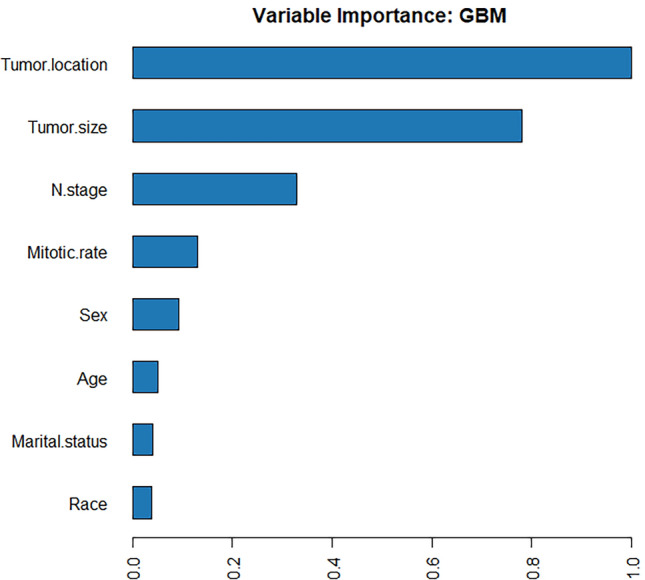
Variable importance of the GBM model in the training cohort, showing that tumor location was the most important feature, followed by tumor size, N stage, mitotic rate, etc.

**Figure 4:**
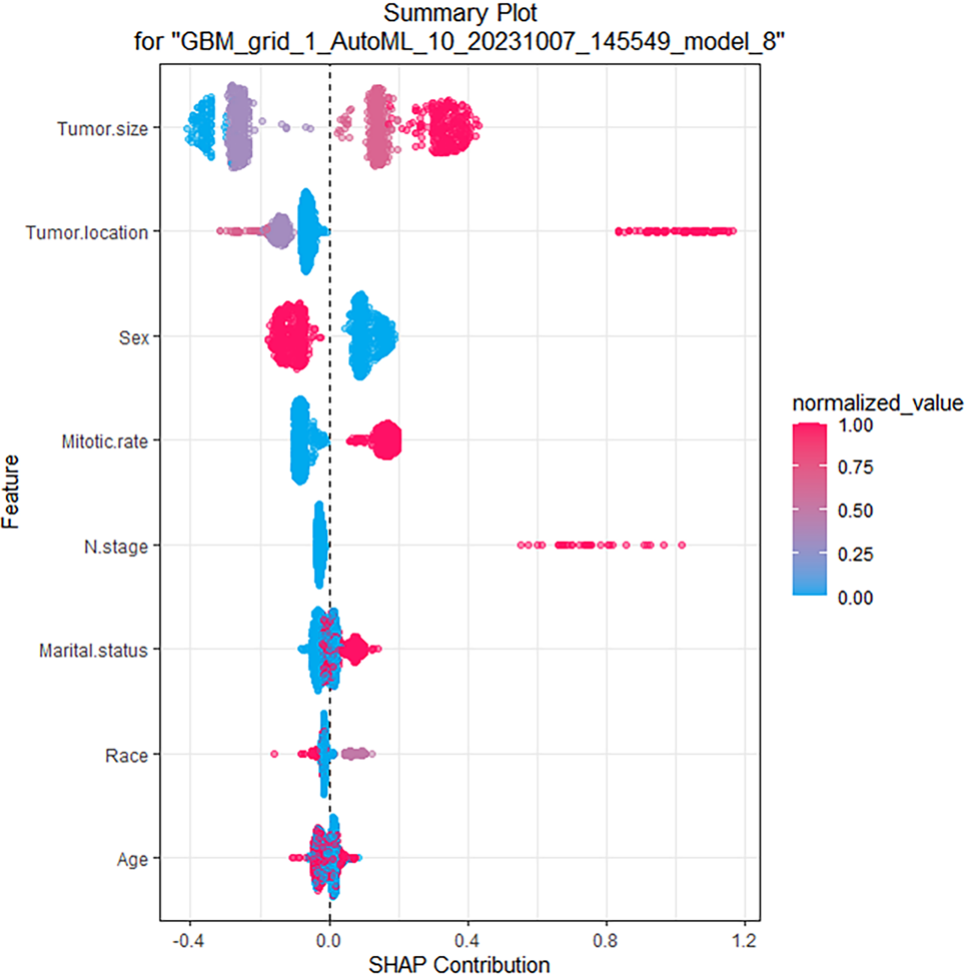
SHAP of the GBM model in the training cohort. As a variable's value approaches 1, the likelihood of a patient developing liver metastasis increases. *SHAP* SHapley Additive explanation, *GBM* gradient boost machine.

A total of 41 models were built using four ML algorithms, namely GBM, DL, and GLM, with stacked ensemble models being excluded due to their limited interpretability. Delving deeper, the GBM yielded 19 models by tuning diverse hyperparameters. The DL method generated 19 models, capitalizing on a range of architectural setups, activation functions, and regularization techniques. Meanwhile, the GLM contributed 3 models, adjusting for variations in family distributions, link functions, and regularization intensities. Among the models built using the four ML algorithms, the GBM model (GBM_grid_1_AutoML_5_20231007_200850_model_6) demonstrated superior performance, achieving the highest AUC values and accuracy. Therefore, it was considered the most optimal model. According to Fig. 3, the most important feature was found to be tumor location, followed by tumor size, N stage, mitotic rate, sex, age, marital status, and race, in that order of decreasing importance. Additionally, the GBM and logistic regression models both identified tumor location and tumor size as important variables in predicting the outcome. The SHAP contribution plots generated by GBM algorithms are presented in Fig. 4, highlighting the eight variables with the greatest impact on the outcome. These variables are tumor size, tumor location, sex, mitotic rate, N stage, marital status, race, and age. When a variable's value approaches 1, the probability of a patient having LIM increases. This is exemplified by the SHAP plot in which the red dots representing tumor size larger than 5.0 cm are primarily situated on the right-hand side of the zero axis, indicating that patients with a large tumor size have a higher likelihood of experiencing LIM.

**Figure 5:**
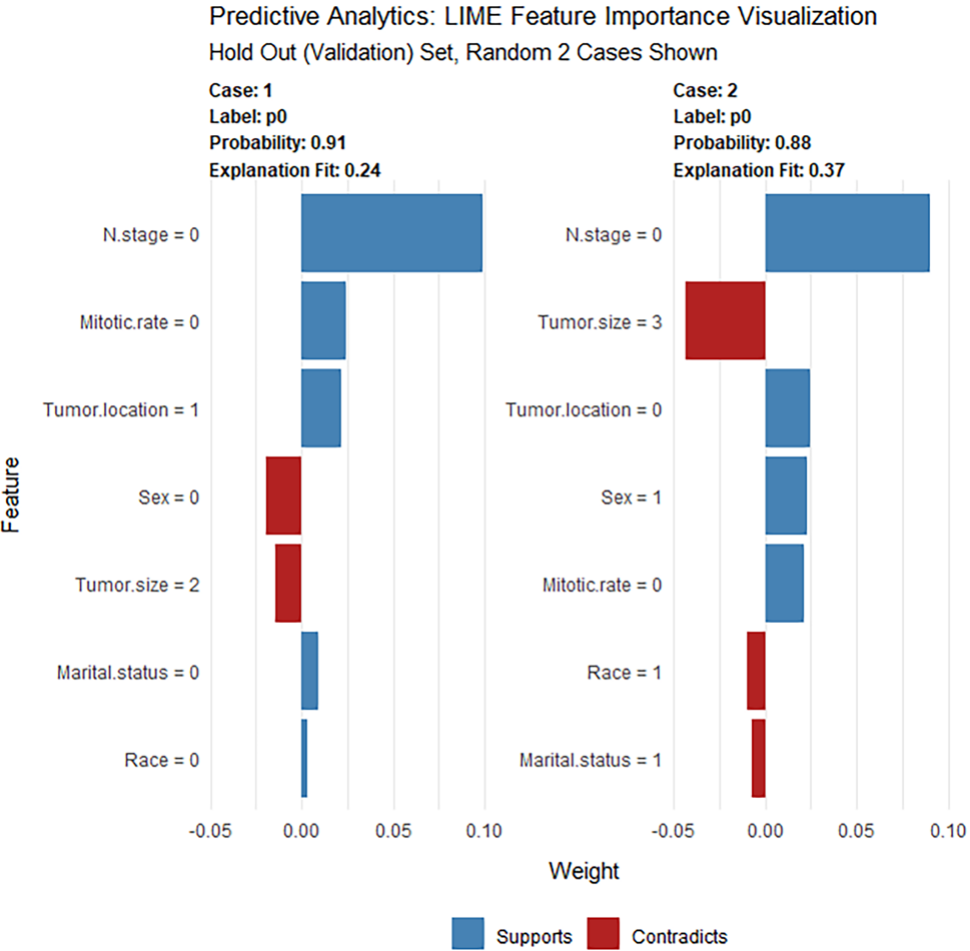
LIME of the GBM model in the validation cohort. *LIME* Local Interpretable Model Agnostic Explanation.

Table 3 indicates that the GBM algorithm demonstrated superior performance compared to the DL, GLM, and LASSO algorithms in the training, validation, and test cohorts in terms of AUC. The GBM algorithm achieved AUC values of 0.805, 0.780, and 0.795, respectively, which were higher than those of the DL algorithm (0.759, 0.729, and 0.746, respectively), the GLM algorithm (0.749, 0.719, and 0.737, respectively), and the LASSO algorithm (0.670, 0.610, and 0.567, respectively). Moreover, the GBM algorithm exhibited the highest accuracy values compared to the DL, GLM, and LASSO algorithms, as shown in Table 3. Figure 5, which features a LIME plot based on the GBM model, highlights the impact of significant variables on LIM of gGISTs. For instance, the GBM model predicted that case 1 in the validation set had a probability of 0.91 for LIM. N stage was deemed the most significant predictor of LIM, followed by mitotic rate, tumor location, maritial status, and race, while the impact of sex and tumor size on these factors was found to be opposite. According to Supplementary Fig. 4, the DCA plots of the AutoML models demonstrated a net benefit of approximately 5%.

**Table 3:** Performance of the machine learning models in the training, validation and test group.

	AUC	Accuracy	Sensitivity	Specificity	F1-score	PPV	NPV
Traing group							
AutoML							
GBM	0.805	0.747	0.747	0.748	0.392	0.266	0.960
DL	0.759	0.701	0.712	0.700	0.340	0.225	0.952
GLM	0.749	0.724	0.658	0.732	0.340	0.231	0.946
Logistic regression analysis							
LASSO	0.670	0.742	0.370	0.787	0.239	0.175	0.911
Validation group							
AutoML							
GBM	0.780	0.700	0.761	0.693	0.343	0.222	0.962
DL	0.729	0.630	0.734	0.618	0.290	0.181	0.953
GLM	0.719	0.607	0.750	0.590	0.282	0.174	0.953
Logistic regression analysis							
LASSO	0.610	0.668	0.402	0.699	0.200	0.133	0.910
Test group							
AutoML							
GBM	0.795	0.706	0.817	0.693	0.371	0.241	0.969
DL	0.746	0.607	0.817	0.582	0.307	0.189	0.964
GLM	0.737	0.681	0.726	0.676	0.327	0.211	0.954
Logistic regression analysis							
LASSO	0.567	0.605	0.419	0.627	0.184	0.118	0.900

*AutoML* automated machine learning, *AUC* areas under the receiver operating characteristic curves, *PPV* positive predictive value, *NPV* negative predictive value, *DL* deep neural net, *GBM* gradient boost machine, *GLM* generalized linear model, *LASSO* least absolute shrinkage and selection operator.

## Discussion

Using the SEER database, a nomogram for predicting GIST liver metastasis was established by Zhou et al.^11^. In reviewing their study, we identified certain variables with "unknown" values, potentially compromising predictive accuracy. To bolster the integrity of our data, mitigate bias, and preserve the original data's variability, we integrated MI techniques to address these missing entries. Simultaneously, leveraging the SEER database, we probed the efficacy of four ML algorithms in predicting the LIM for patients with GISTs. The results demonstrate that the GBM model performs better than other models, with an AUC of 0.780 and 0.795 in the validation and test sets, respectively. In comparison to the nomogram, the AutoML models we constructed demonstrates a higher AUC. Additionally, the GBM model has the highest accuracy among the AutoML models, achieving 0.700 and 0.706 in the validation and test sets, respectively. To sum up, the AutoML model utilizing the GBM algorithm has proven to be a valuable tool in predicting the risk of LIM in GIST patients, ultimately aiding clinicians in creating customized treatment plans.

In the realm of AutoML, ensuring model interpretability is not just important—it's paramount. While many of these models might operate as 'black boxes', tools like SHAP and LIME stand out as keystones to illuminate their predictive processes. Drawing from game theory, SHAP offers a comprehensive perspective on feature significance, whereas LIME delves deeper, providing granular insights into individual predictions by employing interpretable models on strategically altered data^21,22^. In concert, they enhance confidence in AutoML, a crucial element for making well-informed clinical decisions. Guided by this understanding, our research is poised to leverage the strengths of SHAP and LIME to provide a meticulous analysis of model interpretability. The SHAP plot, derived from a holistic data analysis, underscores that cases characterized by extensive tumor sizes, the occurrence of lymph node metastasis, and tumors located external to the gastrointestinal tract are more predisposed to liver metastasis, as shown in Fig. 4. Conversely, the LIME plot presents the liver metastasis likelihood for a singular, randomly sampled case, as delineated in Fig. 5.

In our investigation, both the nomogram and the GBM model's SHAP plot, as well as its feature importance chart, highlighted tumor location as a significant predictor for LIM in GIST patients. We noted an amplified risk of LIM when tumors were categorized as "others," primarily associated with extragastrointestinal stromal tumors (EGISTs). Traditionally, gastric gastrointestinal stromal tumors (G-GISTs) are considered to have a milder biological behavior than small gastrointestinal stromal tumors (S-GISTs), possessing less invasive and metastatic tendencies^23^. Research spearheaded by Miettinen et al.^24^ reported an elevated metastatic risk and tumor-related mortality for S-GISTs, especially when tumors exceed 5 cm in size. Concurrently, Kukar et al.^25^ found a tendency for distal metastasis among younger S-GISTs patients. Yet, our data suggests a similar LIM rate between G-GISTs and S-GISTs in both developing and test cohorts. Intriguingly, when tumors were identified as "others" in the developing cohort, there was a pronounced increase in LIM at 29.6% (102/345). This observation implies a higher propensity for LIM when GISTs are located outside the gastrointestinal domain. The predisposition of EGISTs for LIM could be ascribed to several reasons: (1) EGISTs might manifest more aggressive traits at molecular and genetic levels; (2) Their anatomical positioning could facilitate dissemination via the bloodstream, accentuating liver metastasis risks; (3) The relative obscurity and potential vagueness in symptoms of EGISTs could culminate in delayed diagnosis, thus presenting more advanced stages at detection.

According to the AutoML algorithms conducted in our study, tumor size and mitotic rate were identified as significant factors influencing LIM. Miettinen et al.'s study suggests that tumor size, rather than the mitotic rate, is a risk factor for GIST metastasis^26^. However, both Zhou et al.^11^ and Gaitanidis et al.^27^ found that both tumor size and mitotic rate are significantly associated with a higher risk of LIM, which is consistent with the results of our study. A larger tumor size indicates higher proliferative activity, facilitating the escape of tumor cells and their entry into the bloodstream or lymphatic system, ultimately leading to LIM. Similarly, a higher mitotic rate suggests increased abnormal proliferation, associated with greater malignancy and invasiveness. Faster cell division increases the likelihood of tissue invasion and migration. When the mitotic rate exceeds 5/50HPF, the chances of tumor cells entering the bloodstream or lymphatic system rise, promoting liver metastasis. Nonetheless, further research is required to fully comprehend the underlying mechanisms and related factors.

Our study also elucidated that the N stage serves as a significant risk factor for LIM. In contrast to other solid tumors, lymph node metastasis is exceedingly rare in patients with GISTs, and lymph node dissection is typically not necessary during surgery^28^. However, our findings revealed a heightened likelihood of LIM in cases with lymph node metastasis. Meanwhile, previous studies have consistently associated lymph node metastasis with poorer overall survival rates^29,30^. The propensity of GIST with lymph node metastasis to develop liver metastasis may be attributed to several factors. Firstly, the lymphatic system plays a crucial role in the dissemination of cancer cells. When cancer cells infiltrate and metastasize to regional lymph nodes, there is an increased likelihood of these cells gaining access to the bloodstream or lymphatic vessels, which can then transport them to distant sites, including the liver. Secondly, the presence of lymph node metastasis indicates a more advanced stage of the disease and a higher tumor burden. This suggests that the cancer cells have already exhibited an increased capacity for invasion and dissemination, making them more prone to establishing metastatic colonies in organs such as the liver.

Our study had some limitations. Firstly, this is a retrospective study based on the SEER database, where missing data and biases are inevitable. Nevertheless, we utilized MI methods to tackle the problem of missing data and reduce its influence on the analysis. Secondly, this study utilized the SEER database for analysis, which represents a specific population in the United States. While the study included validation and testing cohorts to evaluate the model, further validation through external cohorts may be necessary when generalizing the findings to other populations or regions. Thirdly, The proportion of patients manifesting LIM is notably low, suggesting potential data imbalance that might compromise the predictive model's accuracy. Moving forward, we plan to balance the dataset by possibly combining oversampling of the underrepresented class (patients with LIM) with undersampling of the dominant class. Additionally, we're contemplating the adoption of methods like the Synthetic Minority Over-sampling Technique (SMOTE) to produce synthetic samples for the minority class, an approach that has demonstrated efficacy in previous research. Despite these limitations, this is the first study to apply AutoML for predicting LIM in GIST, and it demonstrates superior predictive capability compared to previous research. Fourthly, in this study, approximately one-third of the mitotic index values were missing. Despite the application of MI techniques, there remains a potential risk of bias in the predictive models. Moving forward, we plan to implement advanced statistical techniques, including weighted analyses and model adjustments, to further mitigate potential biases arising from the missing data.

In conclusion, this study utilized AutoML algorithms to predict the risk of LIM in patients with GIST. The results showed that the GBM model outperformed other AutoML models in terms of performance and accuracy. Tumor size and tumor location were identified as the most significant predictors for accurately predicting LIM using the AutoML model. This study represents the first application of AutoML in predicting LIM in GIST patients, offering clinicians a valuable tool for developing personalized treatment plans.

### Supplementary Information

Supplementary Information.

## Data Availability

Publicly available datasets were analyzed in this study. These data can be found here: https://seer.cancer.gov/. The datasets supporting the conclusions of this article are included within the article.
