# Supplementary material for: Automated machine learning for predicting liver metastasis in patients with gastrointestinal stromal tumor: a SEER-based analysis
Source: Sci Rep. 2024 May 30;14:12415. doi: 10.1038/s41598-024-62311-9 (PMC11139903; doi:10.1038/s41598-024-62311-9)
Supplement: Supplementary file 1 — Supplementary Information. [file 41598_2024_62311_MOESM1_ESM.docx]

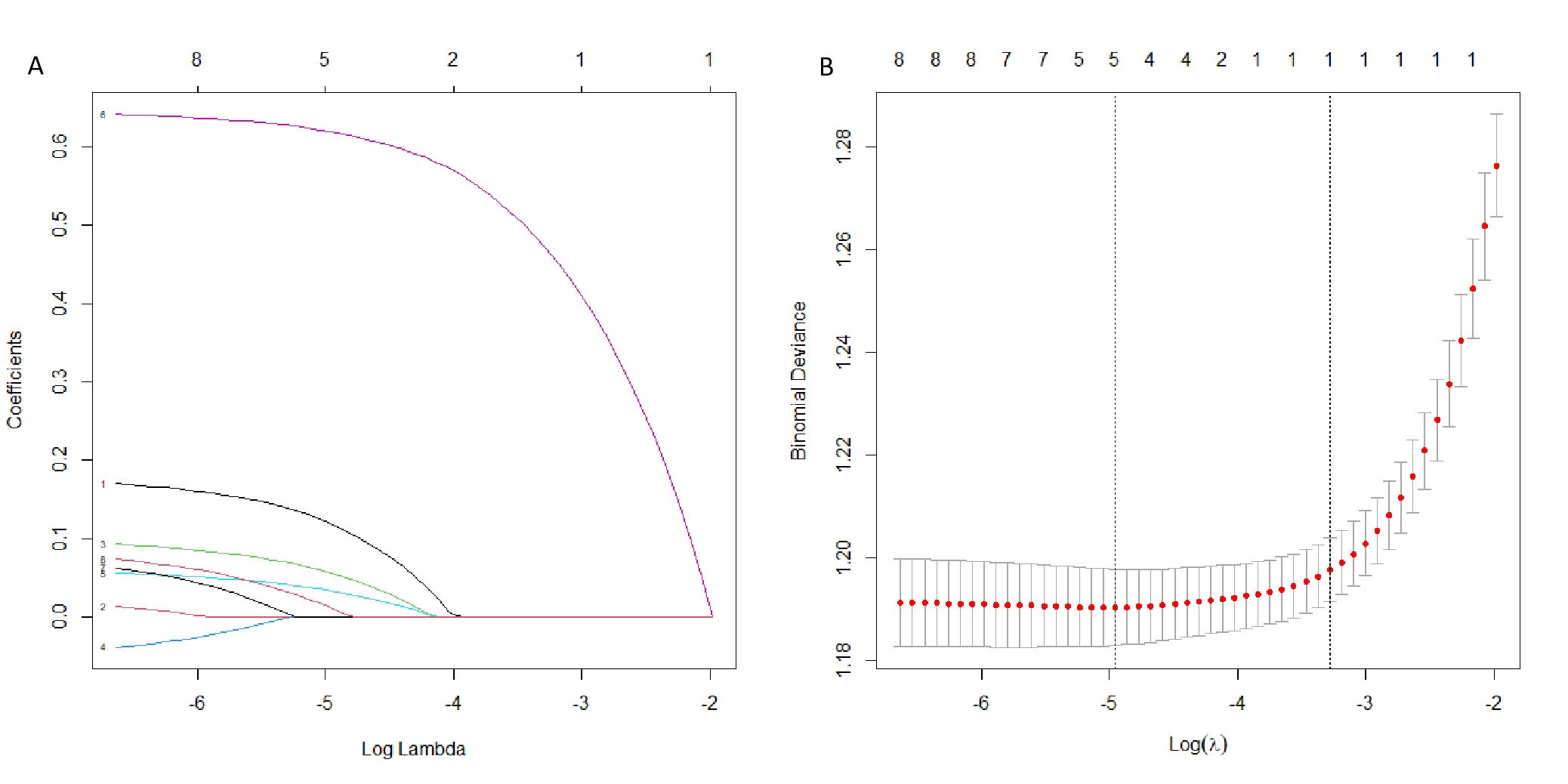


**Supplemantary Figure 1.** Penalty chart of predictive factors for liver metastasis based on LASSO regression analysis. Left: Regression coefficients. With the value of λ increasing, the absolute values of coefficients decrease. Right: Identification of the optimal λ value in the LASSO regression analysis was achieved by 5-fold cross-validation. In our study, LASSO regression model with λ criterion was used in the univariate analysis in order to solve such multiple co-linear relationships among the explanatory variables. LASSO: least absolute shrinkage and selection operator. gGIST: gastric gastrointestinal stromal tumors


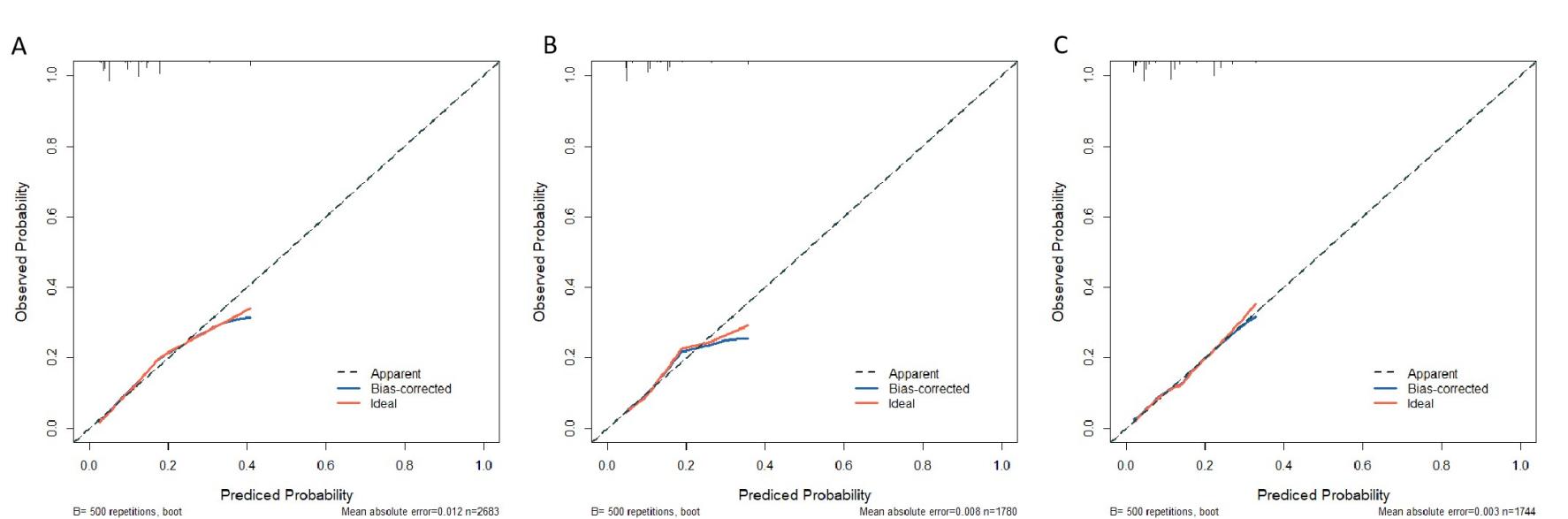


**Supplemantary Figure 2.** Calibration curve of the LASSO model in the training, validation and test set, with the mean absolute errors being 0.012, 0.008 and 0.003, respectively.


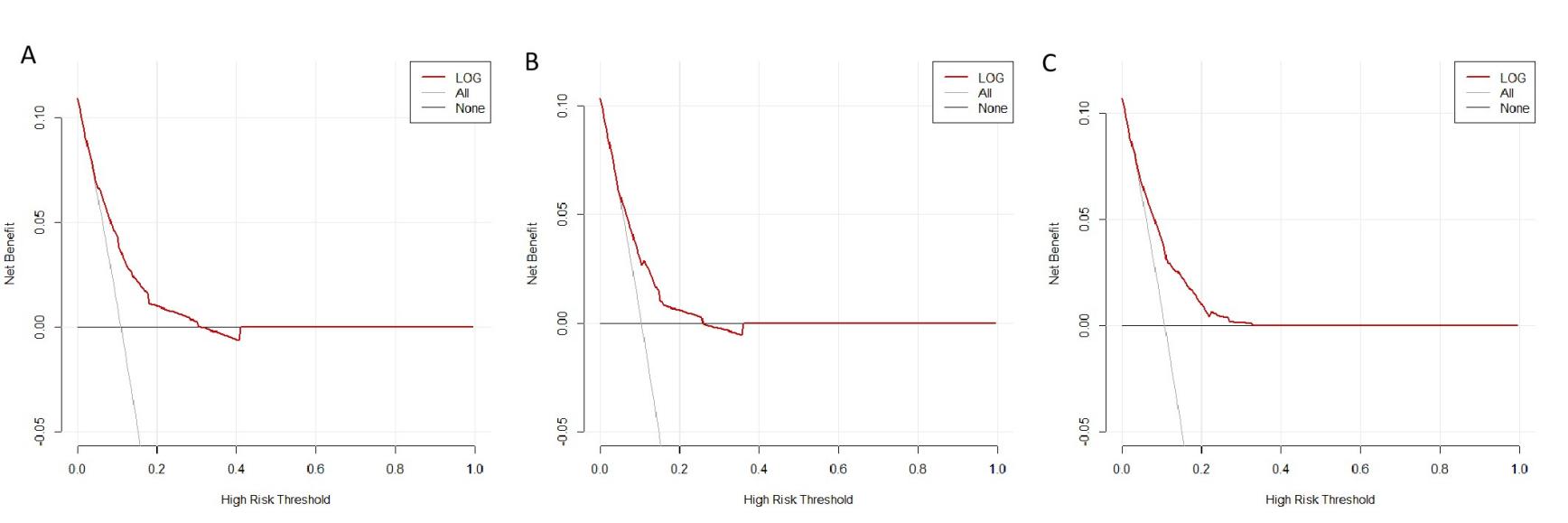


**Supplemantary Figure 3.** Decision curve analysis of the LASSO model in the test set. The DCA plots demonstrated that when the threshold probability of a difficult procedure predicted by the LASSO model was between 10% and 40%, an intervention might add more benefit (0 - 7%).


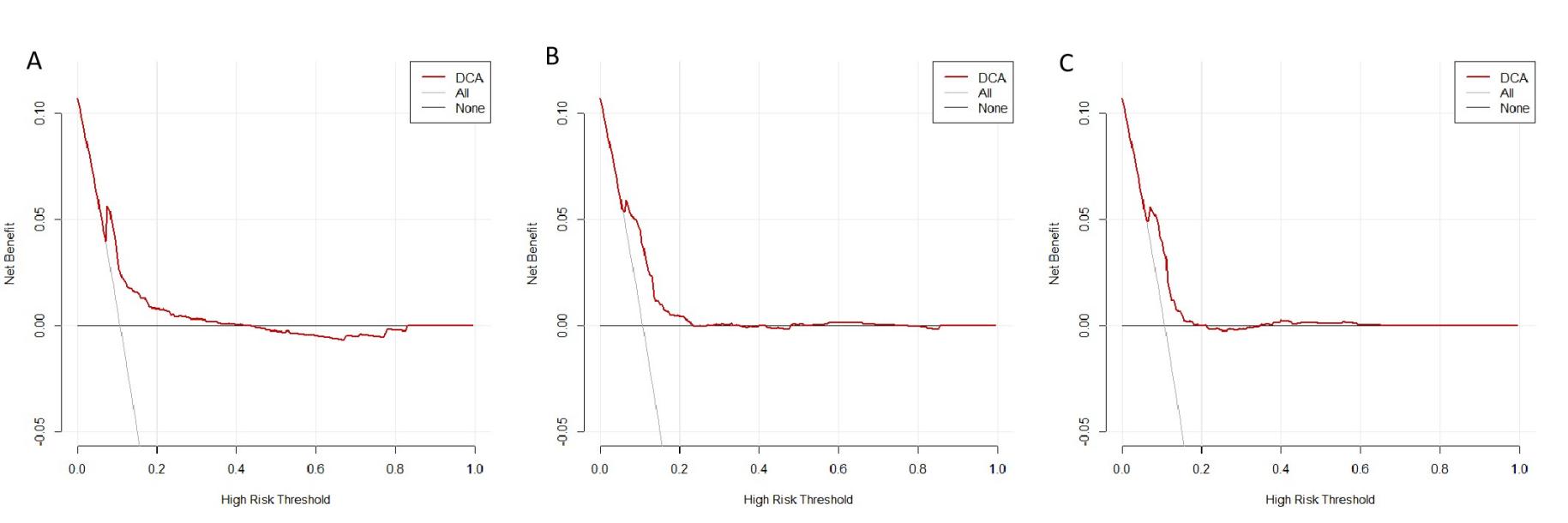


**Supplemantary Figure 4.** Decision curve analysis plots of 3 AutoML models in the test set, indicating net benefits of around 5%. (A) GBM model; (B) DL model; (C) GLM model.

**Supplementary Table 1.** Baseline characteristics of patients before multiple imputation

| Variables | Entire cohort  (n= 6207) | Developing cohort  (n= 4463) | Test cohort  (n=1744) | *P*-value |
| --- | --- | --- | --- | --- |
| Age, years, n (%)  ≤ 65  > 65 | 3221 (51.9)  2986 (48.1) | 2335 (52.3)  2128 (47.7) | 886 (50.8)  858 (49.2) | 0.283 |
| Race, n (%)  White  Black  Others  Unknown | 4172 (67.2)  1130 (18.2)  846 (13.6)  59 (1.0) | 3017 (67.6)  796 (17.8)  615 (13.8)  35 (0.8) | 1155 (66.2)  334 (19.2)  231 (13.2)  24 (1.4) | 0.092 |
| Sex, n (%)  Male  Femlae | 3196 (51.5)  3011 (48.5) | 2301 (51.6)  2162 (48.4) | 895 (51.3)  849 (48.7) | 0.866 |
| Location, n (%)  Stomach  Small intestine  Colon  Others | 3833 (61.8)  1602 (25.8)  290 (4.7)  482 (7.8) | 2748 (61.6)  1162 (26.0)  208 (4.7)  345 (7.7) | 1085 (62.2)  440 (25.2)  82 (4.7)  137 (7.9) | 0.934 |
| N stage, n (%)  N0  N1  Unknown | 5591 (90.1)  216 (3.5)  400 (6.4) | 4067 (91.1)  158 (3.5)  238 (5.3) | 1524 (87.4)  58 (3.3)  162 (9.3) | **<0.001** |
| Tumor size, cm, n (%)  ≤ 2.0  2.0 - 5.0  5.0 - 10.0  > 10.0  Unknown | 821 (13.2)  1830 (29.5)  1693 (27.3)  1380 (22.2)  483 (7.8) | 555 (12.4)  1322 (29.6)  1234 (27.6)  1028 (23.0)  324 (7.3) | 266 (15.3)  508 (29.1)  459 (26.3)  352 (20.2)  159 (9.1) | **0.001** |
| Mitotic rate, HPF, n (%)  < 5/50  ≥5/50  Unknown | 3012 (48.5)  1338 (21.6)  1857 (29.9) | 2077 (46.5)  1001 (22.4)  1385 (31.0) | 935 (53.6)  337 (19.3)  472 (27.1) | **<0.001** |
| Marital status, n (%)  Married  Unmarried  Unknown | 3496 (56.3)  2378 (38.3)  333 (5.4) | 2507 (56.2)  1714 (38.4)  242 (5.4) | 989 (56.7)  664 (38.1)  91 (5.2) | 0.906 |
| Liver metastasis, n (%)  Yes  No | 662 (10.7)  5545 (89.3) | 476 (10.7)  3987 (89.3) | 186 (10.7)  1558 (89.3) | 1.000 |

HPF: high power field; Others (Race): American Indian, Alaska Native, Asian/Pacifc Islander; Others (Location): Tumor locations with fewer than 20 reported cases exist.
